# Pediatric cardiopulmonary resuscitation in infant and children with chronic diseases: A simple approach?

**DOI:** 10.3389/fped.2022.1065585

**Published:** 2022-11-17

**Authors:** Davut D. Uzun, Kristin Lang, Patrick Saur, Markus A. Weigand, Felix C. F. Schmitt

**Affiliations:** ^1^ Department of Anaesthesiology, Heidelberg University Hospital, Heidelberg, Germany; ^2^ Department of Radiation Oncology, Heidelberg University Hospital, Heidelberg, Germany; ^3^ National Center for Tumor Diseases (NCT), Heidelberg University Hospital, Heidelberg, Germany; ^4^ Department of Pediatric Cardiology and Congenital Heart Diseases, Heidelberg University Hospital, Heidelberg, Germany

**Keywords:** resuscitation, cancer, congenital heart failure, cardiopulmonary rescuscitation, chronic diseases

## Abstract

Infants and children with complex chronic diseases have lifelong, life-threatening conditions and for many, early death is an unavoidable outcome of their disease process. But not all chronic diseases in children are fatal when treated well. Cardiopulmonary resuscitation is more common in children with chronic diseases than in healthy children. Resuscitation of infants and children presents significant challenges to physicians and healthcare providers. Primarily, these situations occur only rarely and are therefore not only medically demanding but also associated with emotional stress. In case of resuscitation in infants and children with chronic diseases these challenges become much more complex. The worldwide valid Pediatric Advanced Life Support Guidelines do not give clear recommendations how to deal with periarrest situations in chronically ill infants and children. For relevant life-limiting illnesses, a “do not resuscitate” order should be discussed early, taking into account medical, ethical, and emotional considerations. The decision to terminate resuscitative efforts in cardiopulmonary arrest in infants and children with chronic illnesses such as severe lung disease, heart disease, or even incurable cancer is complex and controversial among physicians and parents. Judging the “outcome” of resuscitation as a “good” outcome becomes complex because for some, life extension itself and for others, quality of life is a goal. Physicians often decide that a healthy child is more likely to have a reversible condition and thereby have a better outcome than a child with multiple comorbidities and chronic health care needs. Major challenges in resuscitation infants and children are that clinicians need to individualize resuscitation strategies in light of each chronic disease, anatomy and physiology. This review aims to highlight terms of resuscitation infants and children with complex chronic diseases, considering resuscitation-related factors, parent-related factors, patient-related factors, and physician-related factors.

## Introduction

Generally, emergency medicine can prove difficult in children and adults due to the high number of various diseases. If children then also have chronic illnesses, they are among the most vulnerable patients. Scientific publications on the emergency care of chronically ill children are mostly only available for diseases with a high prevalence (e.g., diabetes mellitus), for rare diseases they are completely missing. Not only emergency treatment but also symptom treatment are of high importance at the end of life, which is a difficult situation for emergency physicians. Mortality in the United States of approximately 20,000 infants, children, and adolescents from complications such as prematurity, congenital defects, malignancies, and a variety of other diseases is high (1). Pediatric out-of-hospital cardiac arrest (OHCA) is a relatively rare event with an abysmal prognosis. The 30-day survival rate has recently improved but still varies between 5% and 10% (2), there are other studies that reported survival rates of 23%–33% (3). Prevalence estimates of chronic conditions in children range from 10% to 30% (4, 5). Their chronic conditions often affect multiple organ systems (6, 7). Cardiopulmonary resuscitation is more common in children with chronic diseases than in healthy children and the causes of pediatric cardiovascular arrest differ significantly from causes in adult patients. Resuscitation of children presents significant challenges to physicians. Primarily, these situations occur only rarely and are therefore not only medically demanding but also associated with emotional stress (8). The worldwide valid Pediatric Advanced Life Support Guidelines do not give clear recommendations how to deal with periarrest situations in chronically ill infants and children (9). Much more the general measures of resuscitation are addressed in these guidelines. Shockable cardiac rhythms like ventricular fibrillation (VF) or pulseless ventricular tachycardia (pVT) are common in primary adult cardiac arrest (10). Immediate defibrillation without delay is essential for these heart rhythms. Every minute of delay in defibrillation significantly decreases the probability to return of spontaneous circulation (ROSC) (9, 11, 12). There is a potential need for individualized strategies in children with chronic (cardiac) diseases. Secondary cardiac arrest is much more common in childhood (9). This circumstance usually represents the endpoint of increasing clinical deterioration in the context of disease. Most often, secondary cardiac arrest is the result of manifest hypoxia causing myocardial dysfunction. In principle, hypoxia may be caused by respiratory failure with inadequate oxygenation or by hemodynamic instability with consecutive organ hypoperfusion (13). In such situations, the body's own compensatory mechanisms are activated with the aim of ensuring adequate oxygenation of the relevant organs such as the heart and brain. These compensatory mechanisms are limited and result without adequate emergency management in respiratory or circulatory decompensation. Depending on the pathophysiology, respiratory and circulatory failure may be present simultaneously. In the context of pediatric cardiovascular arrest, usually non-shockable cardiac rhythms like asystolia and pulseless electrical activity are apparent (9). For the majority of children who suffer cardiac arrest, the sequence of actions is based on two facts: First, the majority of pediatric cardiac arrests are hypoxic in nature and therefore a focus is on opening the airway and providing rapid high flow oxygenation *via* bag-mask-ventilation. Secondary, the most common cardiac arrhythmia in pediatric cardiac arrest is severe bradycardia, which usually ends in terminal asystole. Only about 10% of pediatric cardiac arrests primarily show shockable cardiac rhythms. Therefore, rapid initiation of Basic Life Support (BLS) seems more important than quick access to a defibrillator (9). If respiratory arrest is recognized in time and there is still cardiac activity, the neurologically good long-term survival probability is 50%. If cardiac arrest has already occurred, a good neurological outcome is much less likely (9). Therefore, the early detection of critical situations and the immediate management of target-oriented measures are given top priority with the aim of avoiding further decompensation. As with any medical emergency, a systematic and prioritized approach increases diagnostic reliability and improves team communication and teamwork. Assessing the critically ill child in an emergency situation is difficult. Complex information is obtained and treatment decisions are made under time pressure sometimes by providers with limited experience with pediatric patients. As soon as a life-threatening problem is detected, it must be treated immediately before the next investigations follow. This is one of the most important components of the systematic approach in the sense of “treat as you go”. Even with the best medical care, cardiac arrest cannot always avoid in children (14). Basic Life Support should be started in all children who are unresponsive and not breathing normally. The main focus is to achieve sufficient oxygenation to protect the brain, hearth and other vital organs from hypoxia (9).

As stated in detail, there is no data available on the procedure in children with chronic diseases. Essential and efficient help at the highest safety level requires innovative concepts of emergency physicians, pediatricians and paramedics regarding a meaningful support of them. For relevant life-limiting illnesses, a “do not resuscitate” order should be discussed early during hospitalization, taking into account medical, ethical, and emotional considerations. Stopping resuscitation in cardiac arrest in infants and children is complex and controversial among physicians and parents (15). During resuscitation, a distinction must always be made between “stopping resuscitation” and “not resuscitating”. For this reason, timely discussions should be held with the patient's relatives about the goal of resuscitation: preservation of life with a good quality of life on the one hand, or a lack of quality of life on the other.

To be distinguished for each person is the outcome of resuscitation: for some the prolongation of life and for others the quality of life is an important outcome. Medical professionals decide patients with comorbidities and chronic care needs are more likely to have an irreversible condition whereas healthy children are more likely to have a reversible condition (16, 17). This article provides a comprehensive overview in resuscitation of infants and children with complex chronic diseases, considering resuscitation-related factors, parent-related factors, patient-related factors, and physician-related factors. In addition, current European Resuscitation Council guidelines in pediatric life support will be discussed.

## Materials and methods

PubMed database was used for literature work. We include studies (case series, guidelines, pediatric-advanced life support guidelines and uncontrolled studies) in which resuscitation of infants and children with complex chronic diseases were discussed. Since the topic of resuscitation of infants and children is hardly found in the literature, as it is quite a critical topic ethically, and there is no proper field of research on it, an iterative approach was used to select appropriate papers. Case reports, especially on palliative care approaches, were also included to provide an overview of the prehospital situation. Emphasis was placed on data from 2010 onward. The Keywords were “resuscitation in infants and children”, “chronic diseases in children”, “do-not-resuscitate”, “presence of parents during resuscitation” or “ethics during resuscitation in infants and children”. We include only English-language papers and excluded all papers if they had nothing to do with resuscitation in infants or children with chronic diseases. Included papers are summarized in Table 1.

**Table 1:** Overview of essential papers.

Author	Year	Study characteristics	Methods	Outcome
Naim et al. (2)	2017	Registry analysis	Data from a resuscitation registry of nontrauma out of hospital cardiac arrest of children younger than 18 years of age, between 2013 and 2015.	Early lay resuscitation may improve survival of an out of hospital cardiac arrest. Improving lay resuscitation in minority communities and increasing lay use of conventional resuscitation can improve outcomes in pediatric out of hospital cardiac arrest (OHCA).
Feudtner et al. (6)	2000	Retrospective cohort study	Population study in a patient cohort from 1980 to 1997 in America. Mortality rates, number of deaths, and age at death were documented for 9 categories of chronic conditions. Outcome: For each of 9 categories of complex chronic conditions, the number of deaths, mortality rates, and age at death.	Almost 25% of the 21,617 deaths during this period were due to complex chronic disease. There was a peak in incidence in infancy, but different age peaks were evident depending on the disease.
Hawkes et al. (10)	2017	Prospective observational study	The incidence, demographic and outcomes of patients who were treated for an out of hospital cardiac arrest between 1st January 2014 and 31st December 2014 in 10 English Emergency Medical Service (EMS) regions.	Cardiac arrest remains a common cause of death. Survival rates are shown to be approximately 10%. There was a positive outcome with early lay resuscitation and early defibrillation if necessary.
Morgan et al. (13)	2021	Review	Review of current medical knowledge and guidelines in pediatric in-hospital cardiac arrest (IHCA) patients.	Despite continued advances in medicine, more than 50% of children with cardiac arrest die before hospital discharge in the United States. Therefore, early recognition of cardiac arrest and early initiation of basic life support interventions, as well as targeted post resuscitation care, is of relevant importance. Further, many recommendations in this topic area are of low evidence level. Efforts should continue to generate good clinical data to improve survival.
Gupta et al. (15)	2019	Registry analysis	Evaluation of do no resuscitate status and assessment of factors associated with it. Patients <18 years of age with an in-hospital cardiac arrest (IHCA) and performed cardiopulmonary resuscitation of at least 1 min were included. This was done between 2006 and 2015.	Patient-, hospital-, and regional-level factors are related to do-not-resuscitate status after pediatric cardiac arrest. For children with incurable chronic diseases, early discussion of do-not-resuscitate (DNR) status may help families and positively influence quality of life and end-of-life care.
Scholefield et al. (18)	2012	Retrospective cohort study	155 Infants and Children admitted to pediatric intensive care units after out of hospital cardiac arrest (2004–2010). Predictive factors for survival to pediatric intensive care unit discharge.	This large study is one of the first to show lactate as a relevant negative predictor of survival of an infant out of hospital cardiac arrest in the pediatric intensive care unit. In this studied collective, 32% (50/155) of children survived to pediatric intensive care unit discharge. The development of an accurate predictive tool would aid study design and prognosis after pediatric out of hospital cardiac arrest.
Tabbutt et al. (19)	2016	Review	The goal is to present the characteristics of children with congenital and acquired heart disease before, during, and after cardiac arrest.	A full understanding of the anatomic, physiologic, and pathophysiologic circumstances is essential to adequately adjust the general pediatric advanced life support recommendations and potentially improve outcomes.
Marino et al. (20)	2018	Review	Literature review of pediatric patients with heart disease who received resuscitation. Existing databases were searched in a broad time frame from 1966 to 2015.	The risk of suffering a cardiac arrest increases due to imbalance between systemic blood flow and pulmonary blood flow. Further, with altered ventricular pressures and volumes, pulmonary hypertension, valvular pathologies. In the end, these pathologies all lead to an imbalance between oxygen supply and oxygen demand, which in the worst case can lead to cardiac arrest. Therefore, a clear understanding of the pathophysiology is of enormous importance.
Lipshultz (21)	2019	Review	The current state of medical knowledge about the causes of cardiomyopathies in children and the useful approaches for early diagnosis were elaborated in a scientific expert consensus.	Overall, a limited data on the diagnosis and treatment of the infantile cardiomyopathies is apparent. Cardiomyopathies in adults show some overlap with childhood pathologies, but in many cases the pathogeneses are different.
Levi et al. (22)	2001	Registry analysis	Over a large time period, cancer deaths and population estimates were derived from the World Health Organization database. Mortality rates for six childhood cancers (renal, bone tumors, Hodgkin's disease, non-Hodgkin's lymphoma, ocular tumors, leukemias were examined.	Mortality from malignancies of the hematopoietic system showed a marked decline from the mid-1960s onward, averaging about 60%. This corresponds to an approximate total of nearly 4,500 avoided deaths per year. The variation for total cancer mortality approached a factor of approximately 3, with following rates: 9.41/100,000 boys, 7.65/100,000 girls.
Bertuccio et al. (23)	2020	Registry analysis	Analysis of data from the World Health Organization database with calculated mortality rates per 100,000 children aged 0–14 years from 1990 to the latest available year in European countries.	Mortality rates in the presence of leukemia occurred in northwestern Europe. European countries located further south had comparatively high incidences. Furthermore, childhood cancer mortality rates have continued to decline steadily. However geographical differences can still be documented. Therefore, treatment regimens should be disclosed worldwide to allow access for all treating physicians.
Marcus et al. (24)	2022	Retrospective cohort study	Data analysis of a present cross-sectional study among bereaved parents of children with chronic conditions who died between 2006 and 2015. The primary outcome was parent-reported suffering of the child in the last 2 days of life.	The majority of children had progressive genetic diseases of the central nervous system. Nearly 33% of parents of children with chronic diseases reported high levels of suffering in their child's final days of life. Parent preparedness was associated with lower perceived child suffering.
Hinds et al. (25)	2005	Prospective clinical interview	Children older than 10 years were asked about any of the following end-of-life decisions: 1. enrollment in a phase I trial 2. do-not-resuscitate order 3. initiation of terminal care The parents, the child, and the pediatric oncologist providing care were interviewed separately using open-ended questions.	The children with cancer recognized that they would be involved in end-of-life decision making. They showed great understanding, especially of the consequences of their decision, and were able to participate in the decision-making process. The most frequently cited factors for decision-making were relationship-based. These findings are primarily inconsistent with existing developmental theories.
Shaw et al. (26)	2011	Review	Is the presence of parents in the context of infant resuscitation relevant to the processing of death?	Of the parents surveyed, nearly 87% would like to be present during resuscitation. Nearly all parents agreed that the decision whether to witness resuscitation should rest solely with the parents. Almost all parents present during the resuscitation of their child would decide so again in retrospect. This suggests that the presence of the parents could be beneficial.
Tripon et al. (27)	2014	Clinical interview	The questionnaire was sent to 550 physicians who participated in a course and they were asked to forward the questionnaire to the nurses as well. General questions were asked about experiences with parental presence during resuscitation of children. In particular, arguments for and against parental presence were evaluated. Soft factors such as moral position were also addressed.	A total of 343 responses were evaluated. 47% of the physicians and 53% of the nurses had already had experience with parental presence during cardiopulmonary resuscitation. The majority of physicians and nurses were reluctant to make decisions regarding parental presence.
Wolff et al. (28)	2010	Retrospective report	Satisfaction survey of parents enrolled in a palliative care program. Choice of end-of-life environment was also surveyed.	After participating in the palliative care program, 69% of parents preferred their sick child to remain at home at the end of life. Before the program was introduced, this preference was present in only 18% of parents surveyed.
Hauch et al. (29)	2021	Retrospective cohort study	Records of the Pediatric Specialized Palliative Home Care related to Emergency Medical Services (EMS) call-outs in Germany.	There were 27 emergency calls (total 172 Patients analyzed) for a total of 20 families. Palliative illness or complications were the most common causes. Despite being connected to an Specialized Palliative Home Care Team, families continue to call 911. Therefore, good collaboration and training should be sought.

## Results

### Resuscitation in children with cardiac diseases

Children with structural or anatomical heart disease have a 10-fold increased risk of cardiac arrest compared to children with a healthy heart. Furthermore, respiratory failure is the most common cause of cardiac arrest in children (30). Furthermore, these children are also at high risk of receiving CPR in an intensive care unit (31). Out of hospital cardiac arrest has been associated with high mortality in pediatric patients with cardiac diseases (18). Medical staff require extensive knowledge of the anatomical, structural and functional aspects of a child's heart defect in order to implement current recommendations at the highest medical level. A large study of 3,437 patients with congenital heart disease showed an incidence of 4.5% over an observational period of 12 years. The majority of in hospital cardiac arrests occurred after cardiac surgery (81.8%) (32). The general ALS recommendations for treatment of cardiac arrest mostly address children with structurally normal hearts. However, acquired or congenital heart disease in children is a major challenge and needs individualized therapeutic approaches in the resuscitation setting. The hemodynamic features of cardiac diseases seem to be particularly important in this context of advanced resuscitation. Best efforts must be made to identify and address the cause of the arrest. Examples include the following:

• Neonates with ductal-dependent circulation
• Newborns with transposition of the great arteries or hypoplastic left heart syndrome
• Neonates with large aortopulmonary shunts
• Infants with unrecognized arrhythmia

### Cardiac arrest in children with pulmonary hypertension

Cardiac arrest can be triggered by pulmonary hypertension as a complication and often leads to initial events. A few circumstances can be imagined as the cause of cardiac arrest:

• Right ventricular dysfunction with failure
• Pulmonary hypertensive crisis
• Abrupt discontinuation of vasodilator medication
• Sedation during medical procedures
• Cardiac arrhythmias
• Pulmonary hemorrhages

Pulmonary hypertensive crisis is a massive life-threatening situation. On the one hand it is very difficult to manage and on the other hand once cardiac arrest has occurred it can be extremely difficult to achieve return of spontaneous circulation (19). In the case of pulmonary hypertensive crisis, therapy includes not only ALS, but also the administration of inotropics and vasopressors. Both improve heart function and coronary blood flow. Furthermore, the prevention of alveolar hypoxia, hypercapnia and acidosis is the focus of therapy. Furthermore, inhalation of vasodilators should be given if possible. In cases of persistent pulmonary hypertension and manifest shock or cardiac arrest, an early decision should be made regarding extracorporeal life support (ECLS) (20). Unfortunately, not all of the above options are available in all areas of medicine, especially in the prehospital area the therapeutic possibilities are immensely limited. Even with an acute intra-hospital exacerbation at a tertiary hospital, not all advanced treatment options, such as ECLS, are ubiquitously available. Even in rich and developed countries in Europe, not all maximum care hospitals are able to perform ECLS procedures in children.

### Cardiac arrest in children with cardiomyopathy and myocarditis

Cardiomyopathies in children account for a large proportion of mortality. Mortality is largely dependent on the type of cardiomyopathy. The highest mortality was seen in children with pure hypertrophic cardiomyopathy with inborn errors of metabolism; here, the estimated rate of death as well as heart transplantation at 2 years was 57%. Cardiomyopathies are responsible for the most frequent heart transplantations in children from the age of one (21). Nevertheless, even after listing the patient to the waiting list for a transplantation, the persisting lack of donor-organs for infants and children is still a mayor limitation. In children with undiagnosed or already persistent cardiomyopathy, relevant hemodynamic decompensation may occur at any time due to external influences, such as periprocedural sedation or acute decompensation of another disease. The use of inotropics and the reduction of the systemic afterload are the most important treatment tools. Recognizing myocarditis in children is a major clinical challenge for treating physicians, because clinical presentation of myocarditis ranges from mild symptoms to heart failure with consecutive cardiogenic shock. The cardiac disease can either heal completely or lead to heart transplantation (33). In early phase of myocarditis, preserved systolic function are often seen on echocardiography. During the disease, there is a high risk of marked deterioration in global cardiac pump function with consecutive long-term consequences. Malignant cardiac arrhythmias such as ventricular tachycardia, bradycardic heart block, and ST-segment changes are common in the setting of cardiac infections. With targeted and adequate therapy, survival in these patient groups is very good. In particular, there is even a very good recovery of cardiac pump function without further residuals (19). For these patients, early initiation of advanced procedures is essential to adequately support compromised myocardial function to allow for full recovery of the myocardium over time. Treatment of children with cardiomyopathies or myocarditis must be provided in specialized pediatric cardiology centers. Even in highly developed countries, this often requires longer transport times to a suitable hospital. Sometimes helicopter emergency service transport is necessary. Long ground-giving transport times or admission to an inappropriate hospital immensely delay the time to effective therapy and should be avoided at all costs. Therefore, rapid diagnosis and timely transport to an ECLS center should be primary (20).

### Cardiac arrest in children with arrhythmia

In children, arrhythmias are less likely to be the cause of cardiac arrest. Causes could be:

• Congenital complete heart block
• Supraventricular tachycardia (SVT)
• Postoperative junctional ectopic tachycardia (JET)
• Long QT syndrome

Congenital complete heart block with normal cardiac anatomic situation is frequently well tolerated. Depending on the rate of the junctional escape rhythm elective pacemaker implantation can be planed in these children. Newborns with congenital heart diseases, particularly complete heart block is generally not well tolerated (34). These children need urgent epicardial pacing, what is technically very demanding due to the size mismatch of pacing devices and the patients’ small anatomy. Temporary pacing can be given in children *via* epicardial wires that remain in place only for a short time. Regular cardiological checks must be carried out frequently.

SVT with the risk of rapid conduction *via* an accessory antegrade pathway is feared. On the one hand, children with Wolff-Parkinson-White syndrome should be observed when they are given drugs that slow down AV nodes, as they can promote stimulus conduction *via* the unprotected accessory pathway (35). Children with SVT and wide-complex tachycardia do not receive adenosine. This meaning is if there is no associated to atrial fibrillation or atrial flutter. In a hemodynamically stable child, electrophysiologic consultation should be obtained as soon as possible. In case of instability synchronized electrocardioversion should be performed urgently. Adequate doses of adenosine can be given in children with narrow complex SVT (36).

JET often occurs as a complication after heart surgery and often presents clinically as complete heart block. For therapy of JET, temporary pacemaker stimulation in AAI mode and drug therapy using amiodarone can be performed as first-line therapy (37). Postoperative JET will heal on its own.

Children with congenital long-QT syndromes are predisposed to malignant arrhythmias. Furthermore, life-threatening torsades de pointe tachycardia may occur in these children. Long-term use of beta-blockers is necessary for the therapy of these cardiac arrhythmias. For some high-risk most children, cardioverter defibrillator implantation is also essential. If long QT syndrome is known, all drugs that affect QT time should be avoided. Especially the administration of amiodarone and procainamide should be avoided. If these drugs are prescribed incorrectly, there is a high risk of malignant cardiac arrhythmias and even cardiac arrest. Analysis of data from a pediatric resuscitation registry showed superiority by the use of lidocaine in terms of 24-h survival and rate of ROSC in the presence of shockable rhythm such as VF or pVT (38, 39).

### Resuscitation in infants and children with cancer

In Europe each year approximately 1.800 pediatric patients die of cancer (22, 23, 40). The progressive disease situation is a trigger for premature death of infants and children. Approximately half of the patients in question have a present statement that addresses the issue of resuscitation (24, 25, 41). Decisions at the end of an affected parent's life are the most difficult to make in the context of advanced disease. Data on decision making and parental education are sparse in the literature (24).

There is evidence in the literature that parental decisions impact both the family itself and the emotional well-being of the child (24, 42).

Since the last phase of life of a child suffering from cancer is one of the greatest challenges for medical professionals as well as parents and all family members, it makes sense to deal with the topic of resuscitation as early as possible. Concepts established here make it easier for both sides to act in these tragic situations.

In order to avoid confusion between relatives and medical staff, timely discussions should take place regarding therapy limitation and therapy wishes. Only in this way a clear common line can be taken in decision-making processes. In highly palliative situations when a child has advanced cancer and the damage after resuscitation is often irreversible, it is necessary to familiarize the family promptly and intensively with the concept of not resuscitating the child.

Avoiding unnecessary interventions makes more sense than “going to the end” and thus prolonging suffering. The child's medical record should include a written statement from the parents and their wishes regarding resuscitation (42). In a paper by Mc Callum et al., it was shown that in 77 pediatric patients with cancer and other life-threatening diseases, the median time from DNR to death was less than 24 h. Furthermore, in 8% of cases, there was no DNR order from the families and also treating physicians. The most frequent deaths in the relevant patient population were detected in the intensive care unit. Thirteen patients actually died at home (43). In the work of Wolfe et al. it was found that physicians recognized the progressive tumor disease already 206 days before the death of a child, whereas parents realized this tragic state only 106 days before the death of their own child (44). Prompt and intensive discussions between parents and physicians before the onset of the terminal phase enabled families to cope better with the situation. If the written consent of the parents is not available in a timely manner and the condition worsens when the tumor disease is far advanced, it may be advisable to start resuscitation. Artificial respiration should also be started in this case. In such a situation, however, it is indispensable to have a prompt emergency discussion with the families, the pediatric oncologists, in order to then decide by joint consensus to discontinue resuscitation measures and also any intensive care measures that may have been performed. However, if the parents do not give their consent to discontinue life-sustaining therapy, the full spectrum should be continued (24).

### How does ethics relate to cardiopulmonary resuscitation?

The ethical issues related to resuscitation are various and should be familiar to advanced life support professionals. Because the time for decision making during CPR is very short, professionals must be prepared in advance to choose between ethically difficult alternatives (45). A multidisciplinary team is essential to the treatment of the child and their family members at the end of life. Decisions about resuscitation and therapeutic interventions at the end of life are complex and require medical and interpersonal skills. Establishing goals of care, such as hope, concern, questioning treatment steps, is best accomplished by asking open-ended questions in a trusting atmosphere and through collaborative communication (6, 46). Resuscitation efforts in children with chronic illnesses should focus not only on regaining life and appropriate functions, but also on life without neurologic deficits. Another issue that has been discussed ethically several times in the literature is the presence of parents during resuscitation. Most parents wish to be present in the event of resuscitation. This gives them an overview of the procedures and also gives them the opportunity to say goodbye to their own child. This has been shown in the literature to be a useful way of processing the grieving process (26). If parents are present during the resuscitation process, it is important to inform the parents about the individual processes taking place by the medical staff involved. Within emergency teams, there are different views and conflicting opinions regarding the presence of parents during resuscitation of the child. Some discuss that the presence of family members poses risks, such as interfering with resuscitation procedures, stress on the response teams, and psychological trauma for the parents. Others discuss that the presence of parents interferes with their professional performance and therapeutic decision making or others are afraid of legal consequences if parents observe “mistakes” during resuscitation (27, 47, 48). In case of children, parents must be involved in the decision-making process regarding a DNR order. It is necessary to understand parents’ goals and values. The parents have the right to make decisions regarding the treatment or therapy limitations. In the case of advanced, highly palliative tumor diseases and advanced cardiac disease in a child, the topic of resuscitation should be discussed with the parents and the medical team. Especially when it appears senseless and inhuman (49). However, if there is no medical indication for resuscitation, the medical staff is not obliged to take life-prolonging measures if these are not indicated. However, discussions should take place with parents and no decision should be made without their consent. Nowadays, religious beliefs often lead to conflicts between parents and doctors (50, 51). Keeping a child with metastatic cancer and respiratory failure on a ventilator temporarily because continued life is the highest value to the parents may be temporarily reasonable, especially when there have been no clear statements from parents in acute emergency situations of chronically ill children that included a DNR order. Nevertheless, the infaused prognosis should then be discussed together with physicians and parents. If a common consent is not possible, the local ethics committee should be involved as an impartial mediator. It is important that parents are shown at all times that the child is treated with appreciation. There is much evidence in the literature that a concept of incorporating palliative care from the earliest stages of the child's illness is useful and parents should clearly clarify their intention to initiate or forgo resuscitation of their child (35). Ethics committees of the respective hospitals can support physicians when it comes to end-of-life decisions, also as mediator between the parents and the medical team in cases of disagreements according to the treatment strategies.

### Challenges for palliative home care teams

Nowadays, care for chronically ill children often also takes place in the home environment of the patients and their families. Here, the children can be intensively cared for by specialized nursing staff in the presence of their families in their own homes. In this context, invasive therapies such as long-term ventilation of the children are frequently encountered. An increasing number of so-called specialized palliative home care teams (SPHC) are also available for the care of children with life-limiting illnesses. These teams usually consist of a multi-professional team and accompany the children and their families at regular intervals after their discharge from the hospital. Fortunately, the number of such teams has been steadily increasing in recent years (28). Children who receive home-based palliative care connection often suffer from rare chronic diseases. Due to their complexity, these diseases often pose great challenges for the emergency services. Therefore, the assessment of the overall situation of the child and the accompanying circumstances including the prognosis in such time-critical situations is almost impossible for the emergency physicians. This is a clear advantage for the SPHC teams as they already know the children and their families as well as the respective disease and therapy goals in more detail. An emergency physician summoned in an emergency situation cannot grasp this complex situation within a few minutes. The primary goal of palliative on-site therapy is to limit the burden of symptoms and to provide emotional relief for the entire family. This leads consecutively to a relief and stabilization of the situation for the entire family of the sick child (29).

### Strengths and limitations of the literature and this review

The approach used in this review may be of assistance to those dealing with the difficult issue of resuscitating infants and children with chronic illness. In summary, the wishes of the parents should be included in the medical decision. Unrealistic expectations are problematic, but unrealistic hopes from a psychological perspective are not. A decision about resuscitation measures can be helpful through joint decision-making processes between the parents and the medical team (26, 46–48). There are no clear guidelines for a DNR order in children with chronic diseases. The limitation of this review is the lack of literature on resuscitation measures in chronically ill children. Furthermore, in discussions between doctors and parents regarding a DNR order, the socio-cultural factor must always be included, which includes ethnic groups, races and beliefs of the different nations of origin.

## Future directions

The Review's summarized and listed literature goes beyond simply recommendations for DNR decisions. This is due to the complexity of the topic, particularly for children.

The therapy of children with chronic diseases is a great challenge especially for emergency physicians. Efforts should be made to provide these individuals with specialized training in these areas. This should be an integral part of their training and addressed through regular continuing education. Close and good cooperation between emergency physicians and palliative physicians is therefore essential. In the future, especially for emergency situations, some kind of differentiated and individualized treatment decisions should be considered for chronically ill children. Prospectively, a design could be chosen for relatives and patients that includes, for example, emergency situations with important emergency measures and contact data for doctors and clinics. It would also be conceivable to develop an “app” for relatives of chronically ill children that provides helpful tips, suggestions and assistance in the event of an emergency. Traditionally, healthcare worldwide has been focused on treating acute illnesses. In the case of chronically ill patients, especially in the vulnerable group of children, there are a multitude of care needs that must be met. However, especially here, when patients do not belong to an outpatient palliative care setting such as SAPV (specialized outpatient palliative care), there is a lack of coordination of the treatment chain across providers and sectors. Therefore, in the future, there should be approaches that care for chronically ill children and pursue the goal of eliminating this deficit. An example of this would be “integral care” by an interdisciplinary team and strong networking of different disciplines. Otherwise Pediatricians should receive regular training on DNR, ethics and communication. Regularly repeated meetings in a multidisciplinary team should be used for case discussion. Self-help groups for parents of chronically ill children, but also for the children themselves, should be formed.

Because DNR in chronically ill children is often talked about at a very late stage, there should also be support approaches in the future for families who are not yet ready to talk about this difficult topic. DNR orders should be discussed early in the child's onset of illness, as continued life support treatment may no longer be in the child's best interests. Only through joint discussions between parents and medical staff can DNR decisions be made in a meaningful way and facilitate psychological interaction for the affected family members. The DNR decision should never be rushed and there should always be respect for children's lives. Only if the parents’ decision about a DNR order is made promptly and then also in writing, it is also easier for emergency physicians at the scene to make meaningful decisions. It is also important to advise parents of affected children that written documentation regarding DNR orders should be kept close to the child.
